# Knowledge, attitude and application towards fast track surgery among operating room paramedics: a cross-sectional study

**DOI:** 10.1186/s12913-022-08817-2

**Published:** 2022-11-23

**Authors:** Ting Huang, Jingming Wang, Yuanyao Chen, Zhen Ye, Yiwei Fang, Yuze Xia

**Affiliations:** 1 Renmin Hospital of Wuhan University, Wuhan, P. R. China; 2 Institute of Reproductive Health, Tongji Medical College, Huazhong University of Science and Technology, Wuhan, P. R. China; 3 Center for Reproductive Medicine, Tongji Medical College, Huazhong University of Science and Technology, Wuhan, P. R. China

**Keywords:** Enhanced recovery after surgery, Health knowledge, Attitudes, Practice, Nursing, Operating room nursing

## Abstract

**Background:**

Fast track surgery has shown its effectiveness to accelerate recovery and gained acceptance in many operations. However, data for paramedics using fast track surgery are limited in China. The aims of our study are to evaluate the knowledge, attitude and application status of fast track surgery in paramedics and to provide suggestions for the better application of fast track surgery.

**Methods:**

Two Hundred Ninety-one operating room paramedics were investigated by simple random sampling from October 20 to December 20, 2019 time. A self-reported questionnaire was used to collect data with five dimensions: demographic data, cognitive level, knowledge, attitude and application of fast track surgery. Data were analyzed using qualitative and quantitative methods.

**Results:**

19.93% of participants never heard fast track surgery and only 3.32% of participants were very familiar with it. Gender (0.702, 95% CI 0.109–1.294), technical title (0.342, 95% CI 0.126–0.558) and awareness of the concept of fast track surgery (0.471, 95% CI 0.165–0.776) had a correlation with the knowledge level of paramedics towards fast track surgery. In terms of attitude towards fast track surgery, gender (− 1.944, 95% CI -3.830- -0.058), age (0.303, 95% CI 0.021–0.585) and knowledge score of fast track surgery (0.426, 95% CI 0.014–0.838) are related. Half of the paramedics believe the most difficult problem in the application of fast track surgery was the lack of multi-team communication and cooperation.

**Conclusion:**

The knowledge of fast track surgery among paramedics in Wuhan is poor, and some paramedics have a negative attitude towards it. As the attitude is positively correlated with the knowledge, it is necessary to improve the knowledge level of fast track surgery among paramedics in Wuhan.

**Supplementary Information:**

The online version contains supplementary material available at 10.1186/s12913-022-08817-2.

## Background

In the late twentieth century, there was eloquent scientific evidence that strongly supported perioperative optimization [1]. Some evidence-based medicine for perioperative care has shown that many traditional surgical care approaches, such as preoperative intestinal cleansing and forced bed rest, are unnecessary or even harmful [2]. In contrast, Fast Track Surgery (FTS), a novel and valid nursing concept and program, have beneficial effects and outcomes on patients through multimodal approaches, such as pain management, early nutritional support and mobilization [3, 4].

FTS, also called Enhanced Recovery After Surgery (ERAS), was initiated by Henrik Kehlet in the mid-1990s [5] and developed by the ERAS Society [6], has been spread rapidly these years [2, 7, 8]. The term “Fast Track” was first mentioned in the context of improving outcomes after coronary artery bypass surgery [9]. After that, FTS was used for sigmoid resection patients and good clinical effects were obtained [5, 10]. Now FTS is applied in a great deal of surgical care and has yielded gratifying results. For instance, FTS will shorten the length of hospital stay and accelerate the recovery of patients’ physical function in gynecology oncology [11]. After spine surgery, FTS can reduce postoperative pain of patients [12]. Hospital cost will be saved for those liver surgery patients through FTS [13]. Besides, the risk of lung infection in patients undergoing upper gastrointestinal surgery can be lowered by FTS [14]. Interestingly, FTS can reduce the incidence of postoperative nausea and vomiting in bariatric surgery patients [15].

FTS has been gradually adapted to daily routines in medical establishments. As validated by scholars, it can shorten the convalescence and attenuate the postoperative complications of patients to large extent [16]. Nevertheless, there remains a delay in integrating these FTS tenets into clinical practice. Some researchers even strongly hinder against this conception and consider it obsolete [17]. Here are current controversies and concerns over FTS. First and foremost, there is a gap between theoretical evidence and implementation, which is particular frequent with the postoperative components [18]. In the meanwhile, the deficiency of compliance obstructs the interpretation of the effects of perioperative outcomes [19]. Besides, conflicting published recommendations and guidelines also baffled the medical workers [20]. Though consensus guidelines were proposed in such departments as colorectal surgery [21], they have seldom been modified based on current practice [19]. Additionally, some people find that doctors are always manipulated by their clinical experience, cognition level and personal preferences tend to have discrepant opinions towards optimization approaches favored by FTS [22, 23]. Furthermore, the most common outcome determining the efficiency of FTS is the hospital length of stay, which fails to reveal the homely convalescent stay of patients [24]. As the primary aim of FTS is to diminish the postoperative organ dysfunction and promote rehabilitation [2], therefore we still warrant new indexes which reflects the effects beyond length of stay and comprehend factors affecting normal living status after discharging from hospital. Last but not least, research attaching great significance to the paramedics’ grip on a rudimentary understanding of FTS was quite scarce. Hence, more sophisticated analysis concerned is desperately required.

The purpose of the present study is to investigate the knowledge level of FTS among operating room paramedics, their attitude towards FTS, and the current application of FTS as well as recommend measures for popularization and further implementation of FTS.

## Methods

### Participants

Our research was conducted at Renmin Hospital of Wuhan University in Wuhan, China. A total of 291 operating room paramedics were recruited to participate in this survey from October 20 to December 20, 2019. Data were collected online through Wenjuanxing (www.wjx.cn) with a self-reported and anonymous questionnaire. Twenty of the results were rejected because their questionnaires were incomplete. All participants agreed to fill in the questionnaire and participate in the study.

#### Sampling method

We invited operating room paramedics randomly selected from the hospital to participate in the study using a simple random sampling method. According to this sampling method, the knowledge score obtained in the pre-survey was 2.5 points, the standard deviation was 1.75, the maximum relative error was taken as 10% and the confidence level was 1 - α = 95%. The sample size was calculated as *n* = (Ζ_1-α/2_*σ/δ)^2^, in which n represents sample size, Z means standard normal distribution, α represents inspection level (taking 0.05), σ represents population standard deviation (SD) and δ represents allowable error. It has been calculated that a sample size of 191 produced a two-sided 95% confidence interval with a distance from the mean to the limits that was equal to 0.250 when the estimated standard deviation was 1.750.

#### Inclusion and exclusion criteria

Operating room paramedics who worked at Renmin Hospital of Wuhan University in Wuhan, China and agreed to fill out the questionnaire to participate in the study were included. Non-operating room paramedics, or operating room paramedics who did not work at this hospital or did not agree to fill out the questionnaire to participate in the study were excluded.

### Questionnaire

The questionnaire consists of five parts: demographic data, awareness of the concept of FTS and access to FTS knowledge, knowledge of FTS, attitude towards FTS, and application of FTS. “Demographic data” part includes gender, age, working years, education level, technical title and occupation. In “awareness of the concept of FTS and access to FTS knowledge” part, there are 2 one-choice questions to judge the degree and the way of understanding FTS by operating room paramedics. “Knowledge of FTS” part has 7 one-choice questions, each with a unique standard answer and each correct answer of these 7 questions represents one knowledge score of FTS. “Attitude towards FTS” part includes 9 single choice items, each with five options: totally disagree, slightly disagree, not sure, somewhat agree, completely agree (with a score of 0, 1, 2, 3, 4, sequentially) and a higher score means a more positive attitude. “Application of FTS” part consists of an open multiple-choice question and an open answer question. Detailed questionnaire is presented in supplementary material. Based on the actual content of the questionnaire, we conducted a test of reliability and validity on “attitude towards FTS” part.

#### Reliability

The Cronbach’s α coefficient of the questionnaire was 0.815 (supplementary Table 1). Item-total correlations for all items of the scale were positive (supplementary Table 2). As a result, “attitude towards FTS” part of the questionnaire passed the reliability test.

#### Validity

Kaiser-Meyer-Olkin (KMO) and Bartlett’s sphericity tests were used to evaluate the suitability for the factor analysis. The KMO value was 0.812 while the results of Bartlett’s sphericity test were *χ*^*2*^ = 897.190 and *P* = 0.000 (supplementary Table 3). Therefore, it is suitable to make the factor analysis for the questionnaire.

As shown in supplementary Table 4, the questionnaire consisted of two sub-dimensions and the factor loads of the items varied from 0.506 to 0.852. All the factor loads were over 0.500. The explained variance percent was 38.409% for sub-dimension 1 while 19.465% for sub-dimension 2, and the total explained variance percent of the questionnaire was 57.874%. Therefore, “attitude towards FTS” part of the questionnaire passed the validity test.

### Statistical analysis

Sample size was calculated using PASS (Version 15.0.1). For the questionnaire, reliability and validity tests and data analysis were performed using IBM SPSS Statistics for Windows (Version 25.0). Mean ± SD was employed for the continuous variables, such as age, working years, and attitude scores, whereas frequency and percentage were used for categorical variables, such as gender, education level, and access to FTS knowledge.

Multiple linear regression was used to establish the potential characteristics related to FTS knowledge score and FTS attitude. *P values* < 0.05 (α = 0.05) indicated that a difference was statistically significant.

Forest maps were constructed by GraphPad Prism (Version 8.3.0).

## Results

### Characteristics of participant

The average age and working years of the participants are 28.56 ± 6.35 and 6.33 ± 7.00 respectively. The participants tended to be female (84.87%), an educational level of undergraduate or higher (79.34%), a junior technical title (53.87%), and heard but known a little about the concept of FTS (60.52) (Table 1).

**Table 1:** Socio-demographic characteristics of operating room paramedic

Characteristics	Total (***n*** = 271)	Percent (%)
**Gender**		
Male	41	15.13
Female	230	84.87
**Age, mean ± SD (year)**	28.56 ± 6.35	/
**Working years, mean ± SD (year)**	6.33 ± 7.00	/
**Education level**		
Less than college degree	56	20.66
College degree and higher	215	79.34
**Technical title**		
Junior	146	53.87
Intermediate	11	4.06
Senior	81	29.89
Unknown	33	12.18
**Occupation**		
Primary nurse	150	55.35
Head nurse	79	29.15
Others	42	15.50
**Awareness of the concept of FTS**		
Never heard	54	19.93
Heard, known a little	164	60.52
Familiar with FTS	44	16.24
Very familiar with FTS	9	3.32
**Access to FTS knowledge (*n*** **= 217**^**△**^**)**		
Special lectures in hospitals	117	53.92
Continuing Education	60	27.65
the Internet	64	29.49
Books or publications	55	25.35
Others	12	5.53

^†^*SD* standard deviation, *FTS* fast track surgery

^△^Only 217 operating room paramedics have heard fast track surgery

### Access to FTS knowledge

Among 217 participants who have heard the concept of FTS, 53.92% had participated in special lectures in hospitals, 27.65% had participated in Continuing Education, 29.49% had obtained FTS knowledge through Internet, and 25.35% had read books or publications about FTS (Table 1)**.**

### Knowledge of FTS

The questionnaire contains 7 knowledge questions (K1-K7) related to FTS (Table 2). Specifically, 177 (65.31%) participants know “K1: What does FTS include?”, 164 (60.52%) participants correctly answer “K2: What are the preoperative preparations recommended by FTS?”, and 165 (60.89%) participants correctly answer “K6: How does FTS recommend to reduce the intraoperative stress of patients?”. However, the correct rate of four questions, “K3: How does FTS recommend bowel preparation before surgery?”, “K4: How long does FTS recommend fasting before surgery?”, “K5: How long does FTS recommend to refrain from drinking before surgery?”, and “K7: How does FTS recommend pain management?”, were no more than 35%, especially K4 and K7 less than 10%. It indicates that the participants tended to be relatively poor in the knowledge of preoperative bowel preparation and pain management recommended by FTS.

**Table 2:** Seven knowledge questions (K1-K7) related to FTS

Knowledge questions	Total (***n*** = 271)	Percent (%)
**K1: What does FTS include?**		
True	177	65.31
False	94	34.69
**K2: What are the preoperative preparations recommended by FTS?**		
True	164	60.52
False	107	39.48
**K3: How does FTS recommend bowel preparation before surgery?**		
True	94	34.69
False	177	65.31
**K4: How long does FTS recommend fasting before surgery?**		
True	25	9.23
False	246	90.77
**K5: How long does FTS recommend to refrain from drinking before surgery?**		
True	61	22.51
False	210	77.49
**K6: How does FTS recommend to reduce the intraoperative stress of patients?**		
True	165	60.89
False	106	39.11
**K7: How does FTS recommend pain management?**		
True	17	6.27
False	254	93.73
**Average score, mean ± SD**	2.59 ± 1.68	/

^†^*FTS* fast track surgery, *SD* standard deviation

Participants can achieve a knowledge score of FTS for each correct answer to these 7 questions. The number of questions answered correctly by all participants is 2.59 ± 1.68, which means that the average score of participants is 2.59 ± 1.68 (Table 2). We established the association between the knowledge score of FTS and characteristics of paramedics by multiple linear regression, as shown in (Table 3). The results showed that gender (B 0.702, 95% CI 0.109–1.294), technical title (B 0.342, 95% CI 0.126–0.558) and awareness of the concept of FTS (B 0.471, 95% CI 0.165–0.776) had a significant correlation with knowledge score of FTS. Female paramedics have better knowledge than male. In addition, the higher technical title paramedics have and the more familiar paramedics are with FTS, the better they master FTS knowledge.

**Table 3:**
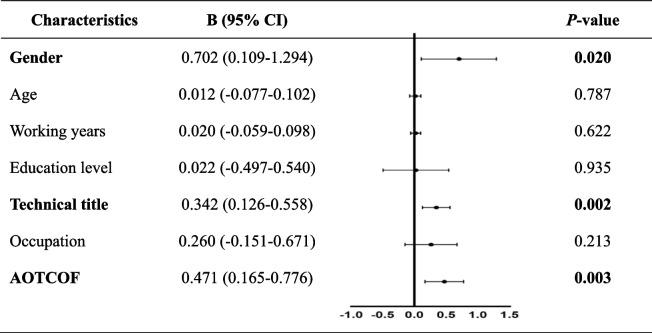
Association between knowledge score of FTS and characteristics of operating room paramedic

^†^*FTS* fast track surgery, *CI* confidence interval, *AOTCOF* awareness of the concept of fast track surgery

### Attitude towards FTS

Totally, 64 (23.62%) paramedics had a negative attitude (attitude scores less than 21) towards FTS; on the other hand, only 52 (19.19%) paramedics had a positive attitude (attitude score more than 30) towards FTS (data not show). We established the association between attitude towards FTS and some potential factors by multiple linear regression model (Table 4). Only gender (B -1.944, 95% CI -3.830- -0.058), age (B 0.303, 95% CI 0.021–0.585) and knowledge score of FTS (B 0.426, 95% CI 0.014–0.838) of participants were related to their attitude towards FTS, indicating that the male and the old paramedics who masters the knowledge of FTS tend to get higher attitude scores of FTS. However, paramedics’ characteristics such as working years, education level, technical title, occupation and familiarity with knowledge, had no significant correlation with knowledge score of FTS (*P* > 0.05).

**Table 4:**
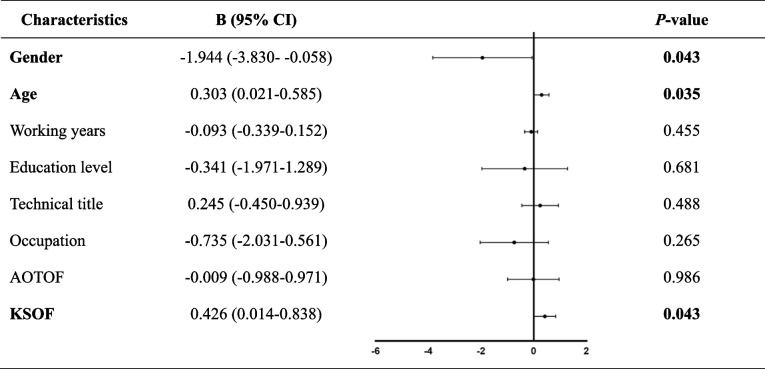
Association between attitude towards FTS and characteristics of operating room paramedic

^†^*FTS* fast track surgery, *CI* confidence interval, *AOTCOF* awareness of the concept of fast track surgery, *KSOF* knowledge score of fast track surgery

### Application of FTS

For the difficulties of FTS in clinical application, 137 (50.6%) paramedics believe the most difficult problem in the application of FTS in the clinic was the lack of multi-team communication and cooperation, 79 (29.2%) believe lack of paramedics, 74 (27.3%) believe that traditional ideas are deeply rooted and difficult to accept changes, and 44 (16.2%) believe lack of evidence-based support. Besides, 14.4% of paramedics believe patient factors. When asked what role can operating room paramedics play in promoting the application of FTS? Some suggest that operating room paramedics should learn more about FTS and share with other colleagues.

## Discussion

As of November 2020, FTS has been propagated and implemented in more than 20 countries worldwide, and China is still in the development stage [8]. This is the first study of the FTS knowledge and attitude of operating room paramedics in Wuhan, China that aims, in part, to understand the current status of FTS nursing knowledge and attitude of operating room paramedics, so as to provide reference for promoting the application of FTS concept. In the present study, 291 participants from Wuhan were enrolled to evaluate their knowledge, and attitudes regarding FTS. In general, the awareness rate of FTS knowledge among paramedics is still at a relatively low level, with no more than half of the FTS questions answered correctly. Our data indicated that operating room paramedics who had senior technical title or were more familiar with FTS, rather than older or longer working years, displayed a high rate of mastering knowledge of FTS. Some of the procedures in FTS, such as early oral feeding, removal of catheters and intravenous transfusion, are often assigned to younger members of the team [25]. Interestingly, female paramedics had a better command of FTS knowledge while male had a stronger identification attitude towards FTS. Moreover, operating room paramedics who were older and mastered the knowledge of FTS better tend to get higher attitude score of FTS. Also, they believed that FTS was necessary and valuable. However, other factors, including working years, education level and occupation, were not associated with mastering knowledge and positive attitudes towards FTS.

Less than 20% operating room paramedics were familiar or very familiar with FTS. Importantly, about 20% never heard the concept of FTS. Furthermore, only 12.9% of the paramedics could correctly answer five or more questions about FTS knowledge. These results show that the paramedics in Wuhan have a low level of understanding of FTS, which might be not conducive to the rapid recovery of patients. Interestingly, even our results showed that the paramedics who had a higher education level or longer working time more likely mastered the knowledge of FTS. Unfortunately, the difference was not statistically significant. But those who have higher education level or longer working time play a significant role in decision-making in nursing practice. Therefore, if they do not understand FTS knowledge, other executives will not be able to complete the content required by FTS well. A survey answered by 272 professionals from 110 hospitals revealed that there is no consensus in Spain, clinicians are familiar with FTS procedures and hospitals do not follow existing guidelines [26].

Recently, clinical guidelines and consensus statements for FTS in many fields have been written and published, such as cardiac surgery [27], gynecologic/oncology surgery [28], lumbar spinal fusion surgery [29] and cesarean delivery [30]. These guidelines standardize the proper application of FTS. However, practical application is difficult and challenging [31]. One of the reasons is that FTS is a multimodal and multidisciplinary nursing program, incorporating surgeons, nurses, anesthetists, and physician assistants [20, 31]. In the meanwhile, FTS includes a lot of complex content which needs the full cooperation of all personnel [20]. There are various obstacles in the implementation of FTS, but the most important ones in this survey are the poor communication and cooperation of multiple teams. As the present study showing, the degree of awareness, knowledge mastery and attitude towards FTS are related. Therefore, it is recommended to publicize and impart knowledge related to FTS in multiple teams, which is more conducive to the rapid recovery of patients. Efficient and perfect application of FTS requires three aspects: great leadership, dedicated coordinator and effective multidisciplinary teams [32]. Jakobsen recommends the workshop-practice method for implementation of new procedures in other areas of patient care [33]. The choice of fast track depends on the instruction, the environment and the sensibility of each surgeon [34].

Although there are several ways for those operating room paramedics to learn FTS knowledge, around 50% of them access to FTS knowledge by special lectures in hospital. Hospitals and relevant departments need to hold relevant FTS knowledge lectures regularly, encourage health worker, especially operating room paramedics to participate, and conduct regular assessments [33].

There is an enormous gap between operating room paramedics’ understanding and practice of FTS, as well as general surgeons [35]. In this study, attitudes of operating room paramedics towards FTS were not very positive, and some even had negative attitudes. To further understand what factor could affect attitudes of paramedics towards FTS, we established a multiple linear regression model. We only found that age and the mastery of FTS knowledge could affect the paramedics’ attitudes to the implementation of FTS, and there were good collinearities. In addition, a survey found that the cognition and attitudes of the medical staff participating in the workshop will be more consistent, which further illustrates the importance of routine workshops for the paramedics in the operating room [34, 36].

## Conclusions

Although FTS has been introduced to China for more than ten years, the implementation of FTS is still in the initial and development stage, so some measures need to be taken to accelerate the application progress of FTS, such as continuing education and training for operating room paramedics. At present, a considerable number of nursing staff are unfamiliar with FTS and hold a negative attitude. The knowledge of FTS was affected by gender, technical title and awareness of the concept of FTS, and the attitudes of operating room paramedics to FTS was affected by gender, age and knowledge. Therefore, it is necessary to improve operating room paramedics’ cognition of FTS, to change their attitudes towards FTS, which is beneficial to its extensive application in China.

We analyzed some factors that affect FTS knowledge and attitude of operating room paramedics. However, the sample size of this study is small and may not be representative of the overall situation in the region. Besides, this research only investigated the problems encountered in the application of FTS and the possible role of operating room paramedics in promoting its application, but did not investigate its current application situation in different hospitals in other regions. In addition, there are few studies on the knowledge and attitude of operating room paramedics and other medical staff towards FTS. In the future, it is necessary to carry out a larger sample of surveys, and further investigate the specific differences and influencing factors of FTS knowledge, attitudes and application in different departments.

## Supplementary Information

**Additional file 1.**

**Additional file 2.**

## Data Availability

The data that support the findings of this study are available from [Wenjuanxing (www.wjx.cn)] but restrictions apply to the availability of these data, which were used under license for the current study, and so are not publicly available. Data are however available from the authors upon reasonable request and with permission of [Wenjuanxing (www.wjx.cn)]. Please contact to Yuze Xia, yzxia07@163.com, if there is any request for the data from this study.
