# Supplementary material for: Knowledge, attitude and application towards fast track surgery among operating room paramedics: a cross-sectional study
Source: BMC Health Serv Res. 2022 Nov 23;22:1401. doi: 10.1186/s12913-022-08817-2 (PMC9685959; doi:10.1186/s12913-022-08817-2)
Supplement: Supplementary file 1 — Additional file 1. [file 12913_2022_8817_MOESM1_ESM.docx]

**Questionnaire on nursing knowledge and attitude towards fast track surgery**

Dear nursing colleagues,

Hello! Thank you for taking time out of your busy schedule to participate in this survey! The purpose of this survey is to understand the current status of operating room paramedics’ knowledge and attitude towards Fast Track Surgery (FTS), also called Enhanced Recovery After Surgery (ERAS), so as to provide reference for promoting the application of FTS concept.

Fast Track Surgery is defined as the use of a series of evidence-based perioperative optimization measures to reduce the physical and psychological traumatic stress of patients undergoing surgery and promote their recovery as soon as possible.

This survey will be conducted anonymously, and the results are for research purposes only. Please fill in your real thoughts and practices, and tick "√" in "□" before the corresponding serial number.

**Thank you very much for your support and cooperation!**

(1) **General information**

1. **Gender:** □ Male □ female

2. **Age: ____** years (full years)

3. **Working years: ____** years (calculated as full years)

4. **Educational level:** □ Less than college degree (technical secondary school & junior college)

□ College degree and higher (Bachelor degree, Master degree and above)

5. **Technical title:** □ Junior (nurse & nurse practitioner) □ Intermediate (nurse-in-charge)

□ Senior (co-chief superintendent nurse and above) □ Unknown

6. **Occupation:** □ Primary nurse □ Head nurse □ Others (Please specify.)

(2) **Awareness of the concept of fast track surgery: (please tick "√" for "□" that meets the description)**

1. **Before this survey, did you hear about Fast Track surgery (FTS)?**

□ Never heard (skip to question K-1) □ Heard, known a little □ Familiar with FTS □ Very familiar with FTS

2. **How did you learn about FTS? (multiple choice)**

□ Special lectures in hospitals □ Continuing education □ the Internet □ Books or publications □ Others (Please specify.)

(3) **Survey of operating room paramedics' knowledge and attitude towards FTS (all are single choice)**

*K part: Knowledge of FTS*

K-1. **What does FTS include?**

□ Anesthesia □ Minimally invasive surgical procedures □ Perioperative care □ Multidisciplinary teams involved in the above three steps (***correct answer***) □ Unknown

K-2. **What are the preoperative preparations recommended by FTS?**

□ Preoperative education □ Nutritional support □ Optimization of organ function □ All of the above (***correct answer***) □ Unknown

K-3. **How does FTS recommend bowel preparation before surgery?**

□ Enema □ Oral laxative combined enema □ No mechanical bowel preparation or only oral laxative (***correct answer***) □ None of the above is correct □ Unknown

K-4. **How long does FTS recommend fasting before surgery?**

□ 12 hours □ 6 hours □ 2 hours (***correct answer***) □ None of the above is correct □ Unknown

K-5. **How long does FTS recommend to refrain from drinking before surgery?**

□ 12 hours □ 6 hours □ 2 hours (***correct answer***) □ None of the above is correct □ Unknown

K-6. **How does FTS recommend to reduce the intraoperative stress of patients?**

□ Optimization of anesthesia protocols □ Minimally invasive surgery □ Intraoperative temperature control □ Fluid management □ All of the above (***correct answer***) □ Unknown

K-7. **How does FTS recommend pain management?**

□ Preoperative preventive analgesia □ Intraoperative optimization of anesthesia protocols □ Early postoperative analgesia (***correct answer***) □ All of the above □ Unknown

*A part: Attitude towards FTS*

A-1. **Is it necessary and beneficial to provide health education and psychological care for patients before an operation?**

□ totally disagree □ slightly disagree □ not sure □ somewhat agree □ completely agree

A-2. **Is preoperative filtration and treatment of malnutrition necessary and beneficial?**

□ totally disagree □ slightly disagree □ not sure □ somewhat agree □ completely agree

A-3. **Can all operations be performed without cleansing enema?**

□ totally disagree □ slightly disagree □ not sure □ somewhat agree □ completely agree

A-4. **Do the harmful effects of traditional methods of fasting outweigh the positive sides?**

□ totally disagree □ slightly disagree □ not sure □ somewhat agree □ completely agree

A-5. **Does controlling patients' temperature during surgery have a significant impact on postoperative recovery?**

□ totally disagree □ slightly disagree □ not sure □ somewhat agree □ completely agree

A-6. **Is the FTS nursing model based on evidence-based medicine?**

□ totally disagree □ slightly disagree □ not sure □ somewhat agree □ completely agree

A-7. **Should the maximization of patients' benefits be the principle before applying FTS?**

□ totally disagree □ slightly disagree □ not sure □ somewhat agree □ completely agree

A-8. **Will the application of FTS increase your workload?**

□ totally disagree □ slightly disagree □ not sure □ somewhat agree □ completely agree

A-9. **Can the FTS nursing model be widely promoted to clinical departments under the guidance of evidence-based evidence?**

□ totally disagree □ slightly disagree □ not sure □ somewhat agree □ completely agree

(4) **Investigation on influencing factors of FTS application**

**What do you think is the biggest difficulty in applying FTS to clinical practice? (multiple choice)**

□ traditional ideas are deeply rooted and difficult to accept changes

□ lack of evidence-based support

□ lack of multi-team communication and cooperation

□ lack of paramedics

□ patient factors

(5) **open-ended question: What roles do you think the operating room paramedics can play in promoting the use of FTS?**
