# Supplementary material for: Knowledge, attitude and application towards fast track surgery among operating room paramedics: a cross-sectional study
Source: BMC Health Serv Res. 2022 Nov 23;22:1401. doi: 10.1186/s12913-022-08817-2 (PMC9685959; doi:10.1186/s12913-022-08817-2)
Supplement: Supplementary file 2 — Additional file 2. [file 12913_2022_8817_MOESM2_ESM.docx]

Supplementary table 1 Cronbach’s α value for “attitude towards FTS” part of the questionnaire (n=271)

|  | **Value** |
| --- | --- |
| Cronbach’s α value | 0.815 |
| Number of “attitude towards FTS” part items | 9 |

^†^FTS, fast track surgery

Supplementary table 2 Explanatory factor analysis results for “attitude towards FTS” part of the questionnaire (n=271)

| **Item** | **n** | **Mean ± SD** | **Item-total correlation** | **If the item is deleted**  **Cronbach’s α value** |
| --- | --- | --- | --- | --- |
| Item 1 | 271 | 3.3690 **±** 0.92516 | 0.589 | 0.787 |
| Item 2 | 271 | 3.3210 **±** 0.84562 | 0.643 | 0.782 |
| Item 3 | 271 | 2.0295 **±** 0.91442 | 0.296 | 0.823 |
| Item 4 | 271 | 2.3137 **±** 0.96674 | 0.379 | 0.814 |
| Item 5 | 271 | 3.1956 **±** 0.91213 | 0.666 | 0.778 |
| Item 6 | 271 | 2.9963 **±** 0.89649 | 0.624 | 0.783 |
| Item 7 | 271 | 2.8192 **±** 1.00026 | 0.561 | 0.791 |
| Item 8 | 271 | 2.5498 **±** 0.86726 | 0.283 | 0.823 |
| Item 9 | 271 | 2.8450 **±** 0.85957 | 0.618 | 0.785 |

^†^FTS, fast track surgery; SD, standard deviation

Supplementary table 3 KMO and BST analysis results for “attitude towards FTS” part of the questionnaire (n=271)

| **Test** | **Value** |
| --- | --- |
| Kaiser Meyer Olkin | 0.812 |
| Bartlett’s sphericity test | *χ^2^*=897.190, *P*=0.000^*^ |

^†^KMO, Kaiser Meyer Olkin; BST, Bartlett’s sphericity test; ^*^*P*＜0.05

Supplementary table 4 Explanatory factor analysis results for “attitude towards FTS” part of the questionnaire (n=271)

| **Item** | **Sub-dimension** | |
| --- | --- | --- |
|  | 1 | 2 |
| Item 5 | 0.852 |  |
| Item 2 | 0.809 |  |
| Item 1 | 0.795 |  |
| Item 6 | 0.778 |  |
| Item 9 | 0.696 |  |
| Item 7 | 0.556 |  |
| Item 3 |  | 0.793 |
| Item 4 |  | 0.768 |
| Item 8 |  | 0.506 |
| (%) Variance explained | 38.409 | 19.465 |
| (%) Total variance explained | 57.874 | |

^†^FTS, fast track surgery
